# The Potential Application of Extracellular Vesicles from Liquid Biopsies for Determination of Pharmacogene Expression

**DOI:** 10.3390/ph15020252

**Published:** 2022-02-19

**Authors:** Henok D. Habtemariam, Henk-Jan Guchelaar

**Affiliations:** Department of Clinical Pharmacy and Toxicology, Leiden University Medical Center, 2333 ZA Leiden, The Netherlands; h.d.habtemariam@lumc.nl

**Keywords:** extracellular vesicles, pharmacogenomics, personalized medicine, exosomes, microvesicles, cytochrome P450, pharmacogene expression

## Abstract

Pharmacogenomics (PGx) entails the study of heritability of drug response. This may include both variability in genes related to pharmacokinetics (drug absorption, distribution, metabolism and excretion) and pharmacodynamics (e.g., drug receptors or signaling pathways). Individualizing drug therapy taking into account the genetic profile of the patient has the potential to make drug therapy safer and more effective. Currently, this approach relies on the determination of genetic variants in pharmacogenes by genotyping. However, it is widely acknowledged that large variability in gene expression is attributed to non-structural genetic variants. Therefore, at least from a theoretical viewpoint individualizing drug therapy based upon expression of pharmacogenes rather than on genotype may be advantageous but has been difficult to implement in the clinical setting. Extracellular vesicles (EVs) are lipid encapsulated structures that contain cargo such as lipids, nucleic acids and proteins. Since their cargo is tissue- and cell-specific they can be used to determine the expression of pharmacogenes in the liver. In this review, we describe methods of EV isolation and the potential of EVs isolated from liquid biopsies as a tool to determine the expression of pharmacogenes for use in personalized medicine.

## 1. Introduction

Pharmacogenomics (PGx) is the study of genetic variation underlying variability in drug response [1]. Specifically, variation in genes that encode for drug metabolizing enzymes, drug transporters, drug receptors or proteins involved in signaling pathways contribute to interindividual variability of drug response. It is now widely acknowledged that variability in pharmacogenes can explain why an individual may experience an adverse drug reaction to a specific medication or could experience inefficient treatment [2]. PGx therefore holds the promise that taking into account an individual’s genotype makes drug therapy safer and more effective.

By genotyping, underlying genetic variation such as single nucleotide polymorphisms (SNP) and copy number variations (CNV) can be determined and the drug dose can be adjusted accordingly [2,3]. Common PGx variants have been described with specific therapeutic recommendations for carriers of certain genotypes and have been presented as a PGx passport, representing close to 50 actionable drug-gene interactions [4]. Alternatively, one may use the phenotype of a pharmacogene to apply personalized medicine. In this approach the so called endophenotype [5], such as drug concentration in plasma or urine following administration of a drug probe, is determined as an indirect measure of drug metabolic enzyme activity. Obviously, determining the endophenotype is more invasive and laborious compared to genotyping and it can only be assessed after administering a (probe) drug.

Moreover, the genotype does not always match the phenotype when it comes to drug response. There are several non-genetic factors and epigenetic factors contributing to the expression of pharmacogenes. Examples of non-genetic influences on pharmacogene expression are gender, weight, age and environmental factors [2]. In addition, there are epigenetic modifications known to alter the transcription of pharmacogenes for example through DNA methylation, histone modifications or miRNAs which are known to be involved in the regulation of the drug metabolism gene family cytochrome P450(CYP450) and drug transporter genes [6]. Therefore, at least from a theoretical viewpoint individualizing drug therapy based upon expression of pharmacogenes rather than on genotype may be advantageous but has been difficult to implement in the clinical setting.

To this end, the use of extracellular vesicles (EVs) isolated from liquid biopsies are of great interest. EVs are structures which are secreted by nearly every cell type and are composed of proteins, lipids and nucleic acids that are representative for their cell of origin. Thereby, the cargo of EVs is protected from degradation by their lipid bilayer [7]. Since EVs are involved in cell-cell interaction, they move through the whole body and are able to influence distant cells and tissues [8]. Liquid biopsies, thus, contain EVs from various cell types making them ideal for biomarker research and phenotyping [9]. In this review, background of EVs and methods for isolation and characterization of EVs from liquid biopsies and their application to determine the RNA and protein expression of pharmacogenes in the liver will be discussed and presented as an innovative method for future application in personalized medicine.

## 2. Extracellular Vesicles

EVs are lipid encapsulated structures that have been conserved through evolution and are found in plants, bacteria and animals [10]. These vesicles were first visualized in the 1950s and were initially considered to be involved in the clearance of cellular debris [11,12]. However, more recent research has shown the involvement of EVs, also, in intercellular communication and development. Through these functions they fulfill a role in determining tissue organization, repair and homeostasis [10,13].

**Figure 1:**
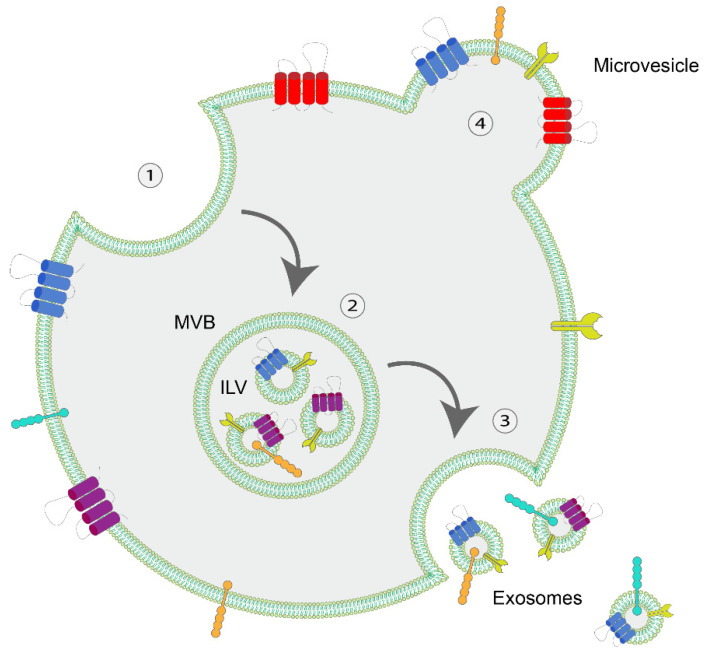
Formation of microvesicles and exosomes. The formation of exosomes by (1) inward budding of the cellular membrane results in the formation of a multivesicular body (MVB) (2) containing intra-luminal vesicles (ILV). After the release of these intra-luminal vesicles the exosomes are formed (3). Microvesicles are formed by outward budding of the cell membrane (4).

EVs can be categorized as exosomes (30–100 nm) or microvesicles (50–1000 nm), and contain cargo such as proteins, lipids and nucleic acids. The cargo is specific to their donor cell although there are various general EV protein markers that are often used as verification of their presence in the sample. These markers are membrane organizers (tetraspanins: CD9, CD81, CD63, TSPAN6, TSPAN8, CD151, CD37, CD53, Flotilin 1 and 2 for exosomes and CD9, CD81 and CD82 for microvesicles), biogenesis factors (Alix, TSG101), adhesion molecules such as integrins and intercellular adhesion molecules (ICAMs) and intracellular trafficking molecules (RAB, GTPases and annexins) [9,13]. Lipids that are identified to be in the EV layer are sphingomyelin, cholesterol, phosphatidylserine, phosphatidylethanolamine and ceramide [9,14]. The nucleic acids that can be found are mRNA, microRNA, siRNA, circRNA, long non-coding RNA and (mitochondrial) DNA [14,15,16,17]. Besides size and cargo, exosomes and microvesicles also vary in the manner that they are formed. Exosomes are formed by inward budding of the cell membrane forming a multi-vesicular body (MVB) in which intra luminal vesicles are formed. After release of these intraluminal vesicles (ILV) they are called exosomes. In contrast, microvesicles are formed by outward budding of the cell membrane [18] (Figure 1). Recent research has revealed variation in morphology between EVs. Electron microscopy has shown much previously unknown variation within Evs showing that Evs can be single, double, double membrane, multilayered or be electron dense [19]. Interestingly, Evs are able to alter the phenotype of their recipient cell by releasing their cargo. In specific EV-cell interaction, vesicles attach to their recipient cells by reciprocated binding to surface receptors. Subsequently, Evs can evoke signaling pathways or be internalized (endocytosis) by the recipient cell. During non-specific interaction the cell membrane simply takes up the Evs through micropinocytosis, phagocytosis or fusion with the membrane [9,14]. Direct fusion of Evs with the membrane results in the release of the cargo directly in the lumen of the recipient cell, while EVs that undergo micropinocytosis or phagocytosis first will come together in the early endosome. The early endosome will be taken up by multi-vesicular body after which the content of the EV will be released or degraded. The EV content can influence both local or distant cells and tissues by autocrine or paracrine communication [20].

Furthermore, EVs are known to be involved in the progression of several pathologies, e.g., the creation of a pre-metastatic niche for cancers and the transport of proteins that are involved in aggregation in neurodegenerative diseases [21]. Moreover, EVs are known to carry pharmacogenomic proteins such as transporters and metabolizing enzymes as well as RNA from pharmacogenes.

### 2.1. Techniques for EV Isolation and Visualization

EVs can be isolated, visualized and characterized through several methods and from various types of samples. EVs can be isolated from cell culture medium, blood, urine, cerebral spinal fluid and breast milk. The isolation and characterization methods are often chosen in line with the specific sample and goal. The most important factors in choosing the method of isolation are yield and purity [22].

**Figure 2:**
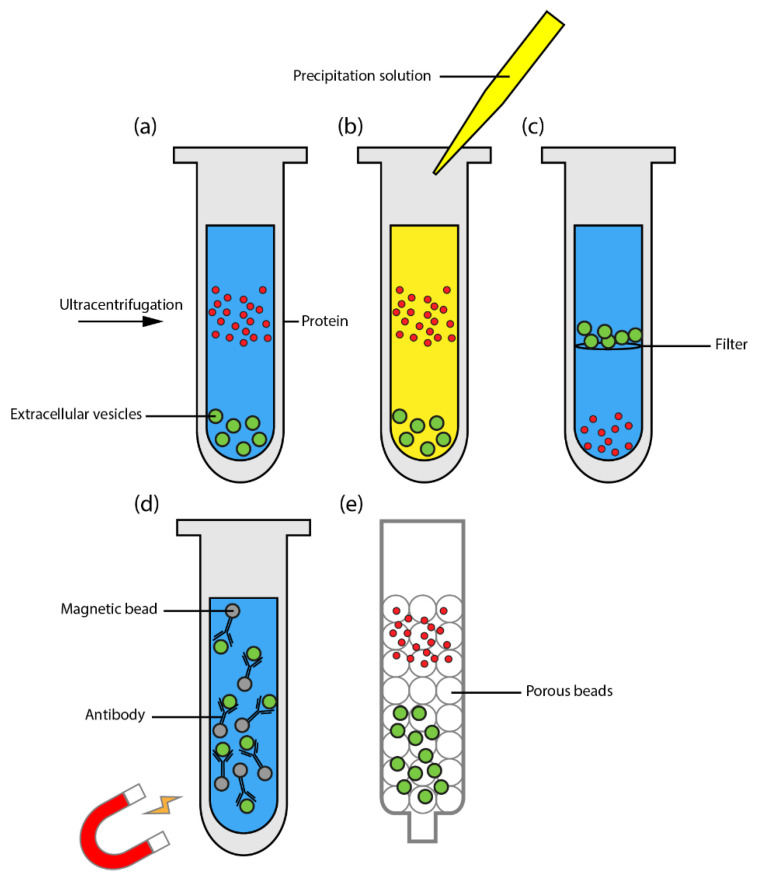
Common EV isolation methods. (**a**). Differential ultracentrifugation is performed at speeds of 100,000× *g* or higher and leads to heavier particles (extracellular vesicles) descending to the bottom to form a pellet while lighter particles (protein) remain in the supernatant. (**b**). Precipitation techniques employ a solution that makes EVs insoluble. (**c**). Ultrafiltration separated particles in a solution based on size. Filters contain a molecular weight cut-off size specific for EV isolation. (**d**). Immunoaffinity methods require an antibody (for example for CD9, CD81 or CD63) conjugated with beads which are upon binding with EVs separated magnetically. (**e**). Size-exclusion chromatography is a technique in which the sample is separated by running through a gel containing porous beads. The sample is separated in fractions with EVs being in earlier fractions and protein in later fractions.

Isolation of EVs can be performed based on size, weight or their composition. Common methods for EV isolation are ultracentrifugation (UC), size-exclusion chromatography (SEC), precipitation, immunoaffinity and filtration methods which can be used in combination (Figure 2) [22].

Ultracentrifugation (UC) is an isolation method that revolves around the weight of the EVs, and is the most commonly used. This method employs centrifugation at high speeds (100,000× *g*) leading to an EV pellet which is subsequently resuspended in a buffer (Figure 2a) [23]. Size-exclusion chromatography (SEC) is a column-based method which can be used manually by kit or by machine and separates EVs based on size (Figure 2e). Precipitation methods aim to make EVs insoluble by using a substance that initiates hydrophobic reactions (Figure 2b) [24]. Precipitation is significantly simpler and requires less time than the UC or SEC methods. Immunoaffinity methods use antibodies that bind to specific EV surface markers making this method highly specific (Figure 2d). The antibodies used for these methods are directed to tetraspanins such as CD9, CD63 or CD81 which are common EV markers. Moreover, several studies have managed to isolate tissue specific EVs by using tissue specific antibodies. Filtration methods employ membrane filters with pores that have a molecular weight cut-off of 10–100 kDa, a size appropriate for EVs [25] (Figure 2c). Prior to performing these methods of isolation, the sample is often filtered or centrifugated at lower speed to remove excess cell debris [26].

Various studies have been performed comparing several common isolation methods based on various parameters. Alvarez et al. [27] compared the yield, purity and RNA levels of several (modified) EV isolation methods such as UC, filtration and precipitation (ExoQuick) using urine samples. The modified ExoQuick method in which they centrifugated at a higher speed showed the highest yield of urine EVs while filtration and UC combined with filtration showed the lowest yield of EVs. The filtration method did show the highest yield in EV protein although the highest yield in miRNA and mRNA levels were seen in de modified precipitation method [27].

Another isolation method comparison was made on EV isolation from serum samples by SEC, ExoQuick (plus), UC and various other methods. This comparison showed the highest yield in the ExoQuick method in particle sizes 0–1000 nm and 0–60 nm which was followed by SEC and UC respectively. Particles 61–150 nm were also most abundant in the ExoQuick method, though UC showed a slightly higher yield than SEC. Furthermore, serum EV protein levels were investigated in which again the ExoQuick came on top, followed by UC and SEC. The particles per µg of protein was highest in SEC followed by ExoQuick and UC [28].

Several EV isolation methods were also compared by Yang et al. [22] who isolated EVs from plasma using SEC, UC, a filtration method (ExoEasy) and a precipitation (ExoQuick) method. The EVs were isolated from plasma with added fluorescent liposomes to determine fluorescent intensity. The study has shown the most fluorescent intensity in the SEC method as well as the lowest protein contamination. The EV size varied between the methods as the ExoQuick contained EVs larger than 100 nm, while in the other methods, the EVs were smaller than 100 nm. Overall, the ExoQuick method showed the highest yield of EVs, however the SEC method showed the highest purity with lowest free protein levels. RNA isolation proved the ExoEasy to have the highest yield of total miRNA compared to the other methods although SEC showed the highest yield of EV-specific miRNA. The highest percentage of mRNA reads was found in SEC after RNA-seq while the ExoQuick presented the highest percentage of long non-coding-RNA. After assessment of RNA-seq data for long chain RNAs, it was revealed that the ExoQuick and SEC method contained mostly exon reads while the other methods contained mostly intergenic reads [22]. Another study compared several precipitation methods in clinical osteosarcoma plasma samples which interestingly showed besides variation in yield, protein concentration and size distribution between the precipitation kits also variation in group specific yield. This means that some methods showed the highest yield in metastatic osteosarcoma condition while with other methods this was similar to control plasma [24].

Overall, the ExoQuick showed to be the best choice if the goal is to have a high yield of EVs in liquid biopsy samples. There were variations in the size and purity between the methods that varied depending on the type of sample.

After isolation EVs are often subjected to a method for EV visualization such as nanoparticle tracking analysis (NTA) and electron microscopy (EM). NTA is most commonly used and utilizes lasers, microscopy and a camera to visualize the size distribution and concentration of EVs [29]. For more specific visualization of EVs the most common method is EM which can show morphological variation in EVs as well as size on a single EV level [19]. Isolation and visualization of EVs is often followed by downstream applications such as methods to research their composition e.g., biomarkers or EVs are modified to use as carriers for biomolecules.

### 2.2. Pharmacogenomic Phenotyping Using EVs

The presence of mRNA and proteins/enzymes encoded by pharmacogenes in EVs from liquid biopsies has been studied and proved in various studies. Initially, a study researching expression in plasma derived EVs, found several CYP enzymes and mRNAs coding for drug metabolizing enzymes such as CYP1B1, CYP2A6, CYP2E1 and CYP3A4. In addition, the study revealed that CYP2E1 and CYP3A4 enzymes from EVs were indeed metabolically active [30]. The presence of metabolic enzymes in EVs has also been proven in vitro. Proteomic analysis showed that rat hepatocyte derived EVs contain several CYP enzymes (2A1,2B3,2C11,2D1,2D3,2D10, 2D18 and 2D26) and UDP-glucuronosyltransferases (UGT) (2B2, 2B3 and 2B5) [31].

The fact that EVs contain pharmacogenomic cargo makes them ideal as a non-invasive method for characterizing an individual’s PGx profile. EVs from liquid biopsies, specifically their cargo, have been researched and utilized in the context of cancer for prognosis and diagnostics but also neurodegenerative diseases, immunological diseases and cardiovascular disease [32]. For instance, in high-grade prostate cancer diagnostics the use of EVs from liquid biopsy, specifically urine, has been validated and uses one cut point making this a binary predictor [33]. In PGx a single cut point would not suffice since we strive to place individuals in a metabolic category thus very specifically predict the appropriate dose of a medication.

Several studies have found creative and innovative ways to solve challenges in developing a PGx expression assay using EVs from liquid biopsies. The first study on the clinical applications of plasma derived EVs in PGx was performed by Rowland et al. [34] who studied the expression of CYP450 and UGT in plasma EVs with the aim to characterize CYP3A4, which is responsible for the metabolism of more than 30% of all drugs, and its induction by rifampicin. They extracted human liver microsomes from tissue samples by centrifugation as well as EVs from plasma samples. The study subjects were selected based on the genotypes CYP3A4*1/*1 (normal activity) and CYP3A5*3/*3 (no activity) [35] eliminating the possibility of drug clearance by CYP3A5. The participants were exposed to an oral dose of midazolam on the first day and the inducer rifampicin daily (until day 8). qRT-PCR confirmed the presence of CYP1A2, CYP2C8, CYP2C9, CYP2D6, CYP2E1 and CYP3A4 and UGT1A1 UGT1A9, UGT2B4, UGT2B7 and UGT2B10 mRNA in the plasma derived EVs. Mass spectrometry revealed the presence of the enzymes CYP 1A2, 2B6, 2C8, 2C9, 2D6, 2E1, 2J2, 3A4 and 3A5 and UGT 1A1, 1A3, 1A4, 1A6, 1A9, 2B4, 2B7, 2B10 and 2B15 in plasma EVs. Additionally, an ex vivo metabolism assay was performed with plasma EVs involving 4-methylumbelliferone (4-MU) glucuronidation and midazolam hydroxylation by UGT1A1 and CYP3A4, respectively. The study showed a great increase in drug metabolism after activation of EVs by alamethicin on ice versus non-activated EVs which was 150 ± 7.6 pmol/min/mg against 6.5 ± 0.4 pmol/min/mg for 4-MU glucuronidation and 14.3 ± 0.7 pmol/min/mg and 0.35 ± 0.7 pmol/min/mg for midazolam hydroxylation. The activity of CYP3A4 ex vivo highly correlated with EV CYP3A4 protein expression (R^2^ = 0.928). Most importantly, a correlation was found between the midazolam clearance and plasma EV CYP3A4 mRNA (R^2^ = 0.79) and protein (R^2^ = 0.90) expression in vivo [34]. Contrary to Rowland et al. [34], Achour et al. [36] used matching plasma EVs and liver tissue to develop a phenotypic assay for use in PGx. They extracted blood plasma and tissue biopsies from liver cancer patients that underwent surgical cancer removal. From the tissue biopsy they isolated healthy tissue which was subjected to mass-spectrometry to estimate the protein composition while mRNA was extracted from plasma EVs and subsequently sequenced. To prove the representability of plasma EV pharmacogene expression, they compared the RNA expression of EVs to protein expression in liver biopsies and found a correlation between EV expression and liver biopsy expression which improved vastly after normalization for individual shedding using a novel shedding factor. The shedding factor was implemented by dividing the EV expression of a gene of interest by the average EV expression of 12 liver specific markers that are highly expressed and consistently detectable in plasma EVs. The correlation prior to adjustment ranged between R^2^ = 0.00–0.53 for CYP450 enzymes, R^2^ = 0.00–0.52 for glucuronosyltransferases (UGT) and R^2^ = 0.04–0.21 for transporters which after adjustment were R^2^ = 0.50–0.75(*p* < 0.001) for CYP450, R^2^ = 0.36–0.65 (*p* < 0.05) for glucuronosyltransferases and R^2^ = 0.43–0.54 (*p* < 0.01) for transporters. A receiver operator characteristic (ROC) analysis, thereby, showed that both the bottom and the top quartile of metabolizers can indeed be predicted with varying accuracies (top quartile AUC ≥ 0.64; bottom quartile AUC ≥ 0.77). Furthermore, an in silico drug trial was performed based on liquid biopsy EV expression using the CYP3A4 metabolized drugs alprazolam, midazolam and ibrutinib. As prior, the categories that were used were the bottom quartile (slow metabolizers), a middle group and top quartile (fast metabolizers). The groups were subjected to three methods of oral administration of medication in the model: a uniform dose, a stratified dose and an individualized dose. After comparison to the uniform dose they showed a 1.7 reduction in variation of drug concentration over time in the stratified condition for all three drugs and in the condition with the individualized dose they showed a 2-fold decrease in variability for alprazolam and midazolam, and a 2.5-fold decrease for ibrutinib. The in silico study confirmed that EV expression based adjustment of drug dose decreases variation in drug plasma levels and could therefore be utilized as a predictor for drug reaction [36].

A novel method for highly specific isolation of liver EVs from a heterogeneous serum sample was introduced by Rodrigues et al. [37,38]. This technique was employed in two of their studies where they studied inducibility of EV pharmacogene expression and drug interactions. They isolated liver EVs from serum by SEC and subsequently an immunoaffinity protocol involving an antibody for the liver enriched marker anti-asiaglycoprotein receptor 1 (ASGR1). In their first research they derived EVs from subjects exposed to oral doses of both midazolam and dextromethorphan (DEX) (day 1) and were daily treated with 300 mg for 8 days or 600 mg for 14 days. The EV samples were assessed prior (day 1) and after rifampicin (day 8 or 15). Midazolam metabolism was found induced as shown by a 72% decrease in midazolam area under the plasma concentration time curve (AUC) after one week of daily 300 mg of the CYP3A4 inducer rifampicin while a two week 600 mg rifampicin use showed a 83% decrease in AUC. Moreover, liver EV CYP3A4 protein expression was significantly increased by rifampicin at both 300 mg (*p* = 0.0005) and 600 mg (*p* = 0.0004). No significant increase was found of liver EV CYP2D6 protein expression as a result of rifampicin treatment. Though, the metabolism of substrate DEX was slightly visible at the 600 mg rifampicin dose, consistent with the fact that CYP2D6 is not or only minimally influenced by rifampicin [39]. They managed to estimate the contribution of CYP2D6 to DEX metabolism by using parameters from previous studies and found a strong correlation between CYP2D6 activity and CYP2D6 liver EV protein expression (r = 0.917, *p* = 0.0001). Lastly, proteomic analysis revealed besides CYP3A4 and CYP2D6 also the presence of CYP3A5, OATP1B1 and OATP1B3 proteins in the liver EVs.

Non-hepatic PGx expression was also studied and compared to hepatic EV PGx expression. They determined the CYP3A4 protein concentration in non-liver EVs which showed an average 2- to 3-fold lower CYP3A4 protein expression than in liver EVs. Though, it is important to note that a higher concentration of CYP3A4 in non-liver EVs than in liver EVs was found in half of the samples. Like the protein CYP3A4 expression in liver and non-liver EVs, there was also variation found in the expression at different doses over time liver EVs showed a mean fold increase of 3.5 at 300 mg rifampicin and 3.7 at 600 mg rifampicin. While in non-liver EVs this was shown to be 2.3 at 300 mg rifampicin and 4.4 at 600 mg rifampicin. These findings suggest that there is individual variability in the dominance of the liver with regards to CYP3A4 expression. Moreover, that the level of CYP3A4 inducibility by rifampicin varies per organ. Besides CYP3A4, a proteomic study was performed revealing expression of both hepatic and non-hepatic CYP and transporters in EVs [37].

Another study by the same researchers was performed on the effects of modafinil as a CYP3A4 EV expression inducer. The participants were genotyped for CYP3A5 and were either CYP3A5*1/*3 (expressers) or CYP3A5 *3/*3 (non-expressers). The plasma samples were obtained from subjects that were exposed to a daily oral dose of modafinil for 14 days. The (endo)phenotype was determined by the plasma concentration ratio of 4β-hydroxycholesterol-to-cholesterol day 1 (pre-modafinil), at day 8 and at day 15. The ratio was increased 1.5-fold and 2.1-fold at 8 days and 15 days after daily modafinil administration, respectively. A proteomic analysis revealed that the liver EVs accounted for 78% of the total EV CYP3A4 expression. Moreover, there was a strong correlation between baseline plasma 4βHC/C ratio and liver EV CYP3A4 with (r = 0.761, *p* = 0.011), and without inclusion of CYP3A5*1/*3 (r = 0.973, *p* = 0.001) carriers. Modafinil showed to significantly increase the protein expression of CYP3A4 1.3 fold (1.1–1.5, *p* = 0.014) in liver EVs, 1.9-fold (1.6–2.2, *p* = 0.04) in non-liver EVs and 1.4-fold (1.3–1.5, *p* = 0.014) globally [38].

The studies managed to find correlations between genotype, phenotype and endophenotype. They unfortunately lacked in the amount of study subjects which ranged between *n* = 5–10 except for the paper by Achour et al. which included 29 participants. Though, in contrary to the other studies Achour et al. did not subject the samples to any genotyping. Overall, these studies demonstrated great potential of using EVs from liquid biopsies for phenotyping the major drug metabolizing liver enzymes.

## 3. Discussion

The use of EVs isolated from liquid biopsies to determine drug metabolizing phenotypes is a novel, innovative and challenging though promising development. Several techniques are being used to isolate these vesicles, with varying results, though UC is regarded the most popular. Comparative studies have studied variation in yield and purity and showed a difference between isolation methods likely also highly dependent on the type of liquid biopsy (urine, serum or plasma). However, studies also showed disconcordances, e.g., a study on isolation methods of EVs from serum found that the particles had a size between 61 nm and 150 nm for several methods (UC, ExoQuick, SEC) but another study on plasma revealed that the ExoQuick gave EVs of 100 nm or above while the other methods (SEC, UC and ExoEasy) gave smaller vesicles (<100 nm). Therefore, it is important to realize that results could vary if research is reproduced using a different EV isolation methodology or kit and thus standardization of isolation methods is required. This is particularly important in the context of PGx, and clinical research in general, as based on the results a clinical decision in the treatment of a patient may be taken.

Currently, the use of PGx has started to be implemented in clinical practice to individualize drug therapy with genotyping techniques being the main method to profile the individual predicted metabolizer phenotype of patients. However, personalizing therapy based upon metabolizer phenotypes as assessed by mRNA expression of pharmacogenes may prove advantageous. Determining the metabolizer status by drug level measurements after administering a drug or probe could be performed but is invasive, costly and laborious. While a few recent studies have clearly shown that phenotyping of pharmacogenes in EVs from liquid biopsies can performed reliably, there have been (besides standardization of isolation methods) several challenges regarding individual normalization and generalization. Achour et al. [36] approached this problem by introducing a shedding factor through which they managed to normalize the pharmacogene expression in EVs by taking into account expression of other common liver markers [36]. A different method was used by Rodrigues et al. [37,38] who specifically isolated liver EVs from serum samples using an antibody for the liver specific marker ASGR1. The advantage of the latter method is the ability to distinguish hepatic and extrahepatic EV enzymes leading to the discovery of the presence of 78% of CYP3A4 in liver EVs and 22% in non-liver EVs. Indeed, effects of both liver and extrahepatic drug metabolism can be investigated in this setting [37,38].

Both the methods of Achour and Rodrigues include a validation procedure as to determine if expression in EVs is a proxy for drug metabolic capacity. In the study of Achour, expression was compared in EVs and matched liver tissue samples. Unfortunately, the study did not provide the genotype of the patients and only a limited distribution of phenotypes was included. Instead, Rodrigues chose to validate by measuring protein concentration of metabolic enzymes in EVs which is obviously a good representation, but does not represent the place in a human being where actual drug metabolism takes place, and thus lacks correlation with liver activity of involved enzymes [40]. Interestingly, they incorporated genotyping and drug concentration ratio measurements in vivo in their studies. While these studies together clearly proved the potential of using EVs in PGx, a more comprehensive validation is needed before clinical application can takes place.

When validated, the method has great potential in PGx. A main characteristic is that longitudinal measurements can easily be performed using liquid biopsies. This enables to study environmental factors and day-to-day variation in drug metabolism. Indeed, variation in drug response or mRNA expression within a specific genotype are often due to phenoconversion, a mismatch between the genotype and predicted phenotype [40]. This mismatch is a result of non-genetic factors such as weight, gender, age and alcohol consumption but could also be disease related for instance infections that lead to the release of cytokines suppressing expression and/or activity of CYP enzymes [40,41].

In addition, the method makes it possible to assess the in vivo functionality of genetic variants of unknown significance. These variants will be determined more frequently in the near future as the result of using sequencing techniques in PGx. In this way, genotype-phenotype translations can be explored more easily and reliably as compared to current methods [42] mainly dependent on bioinformatics predictions. Moreover, the use of EVs in PGx may open novel possibilities to study drug-drug interaction, especially in pharmacokinetic interactions where drugs inhibit or induce metabolic enzymes.

In conclusion, EVs from liquid biopsy can be used to assess drug metabolizing phenotypes, and has, after careful validation, great potential for use in personalized medicine.

## Data Availability

Not applicable.
